# Central Vascular Complications Following Elective Catheterization Using Transradial Percutaneous Coronary Intervention

**DOI:** 10.1177/2324709617698717

**Published:** 2017-03-23

**Authors:** Julia Merkle, Christopher Hohmann, Anton Sabashnikov, Thorsten Wahlers, Jens Wippermann

**Affiliations:** 1 Department of Cardiothoracic Surgery, Heart Center, University Hospital of Cologne, Cologne, Germany; 2 Department of Cardiology, Heart Center, University Hospital of Cologne, Cologne, Germany

**Keywords:** percutaneous coronary intervention, coronary vessel disease, artery dissection, artery perforation, complications

## Abstract

Percutaneous coronary intervention is commonly used to treat coronary artery disease. Both transradial and transfemoral approaches are applied. In general, fewer complications are seen with the transradial approach compared to the transfemoral access, for which reason the transradial catheterization is frequently preferred. In this case presentation, we describe 2 cases of elective transradial coronary angiography both resulting in severe central vascular complications: perforation of the right subclavian artery with a mediastinal hematoma and dissection of the brachio-cephalic trunk and the aortic arch. Although the transradial access is generally considered safe, severe complications such as artery dissection or perforation can occur even in cases of elective procedures.

## Introduction

Cardiovascular disease is a frequent cause of death in Western society. Well-known risk factors are, for instance, obesity, diabetes mellitus, hypertension, or hyperlipidemia. Narrowed coronary artery vessels can lead to an insufficient coronary circulation with typical clinical symptoms. One acute complication of coronary artery disease can be a myocardial infarction necessitating quick intervention. Percutaneous coronary intervention (PCI) is a widely used procedure to treat those obstructions in coronary artery disease and is commonly considered to be safe.

In general, the transfemoral access was shown to be associated with bleeding or vascular complication rate of up to 10%.^1^ The mortality of these complications varies depending on individual patient factors and age.

Large randomized trials comparing the transfemoral with the transradial approach have shown significantly lower rates of vascular and access site bleeding complications with the transradial access without sacrificing procedural success.^2,3^ Thus, the transradial approach seems to be a safer procedure with only scarce description of complications in the literature. Some of the common complications of the transradial access include radial artery spasm, radial artery occlusion, or radial artery injury.^4^

However, as with any invasive procedure, intricacies can occur. Spontaneous coronary artery dissection or artery perforation can be a rare, life-threatening complication of PCI.^5^

In this article, we present 2 cases with significant central vascular complications: perforation of the right subclavian artery and dissection of both the brachio-cephalic trunk and the aortic arch following elective catheterization.

## Case 1

**Figure 1.**
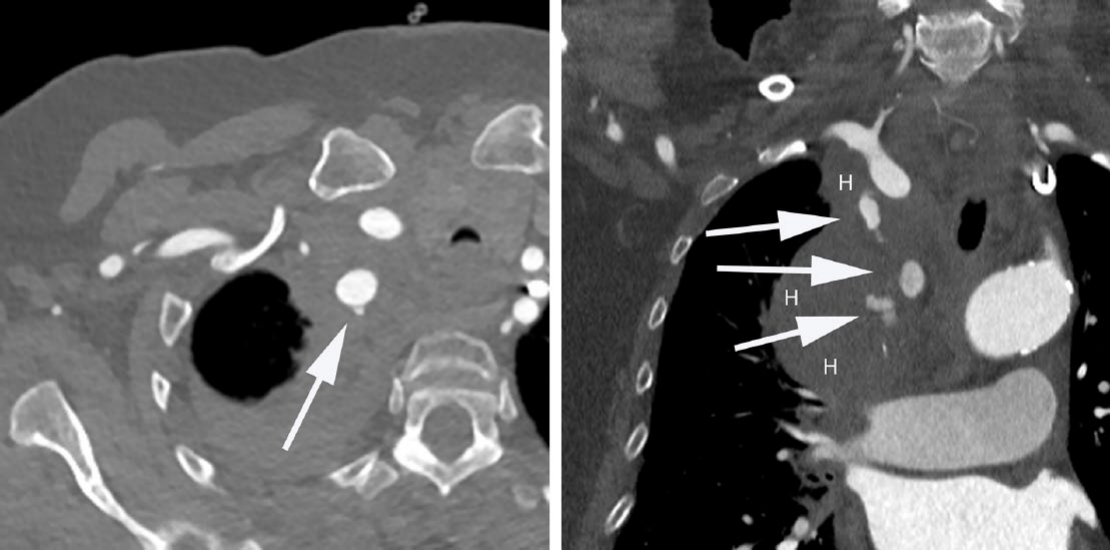
Computed tomography showing perforation of the right subclavian artery (arrows) with a mediastinal hematoma with sagittal and coronal views (H, hematoma).

A 73-year-old woman presented with angina CCS class I. Her past medical history included hypertension, obesity, and an apoplectic event. Electrocardiography did not show any repolarization disturbances, whereas stress echocardiography demonstrated a significant area of inducible myocardial ischemia. Due to these findings and typical symptoms, a diagnostic heart catheterization was performed via the radial artery. A standard dose of 5.000 IE heparin was given. During the procedure the insertion of the polymer-jacketed guidewire was technically difficult probably due to the narrowness of the chosen vessel. The patient complained of back pain. A computed tomography (CT) scan demonstrated a perforation of the right subclavian artery with a mediastinal hematoma (Figure 1), and the patient was referred to the Department of Vascular Surgery for further treatment. Initially, it was decided to treat the finding with a stent. However, during the vascular interventional procedure the leak could not be detected and the procedure was aborted. In a subsequent CT scan the leak was no longer verifiable. Finally, after further conservative treatment the patient was returned to the referring hospital where she was successfully stented via transfemoral PCI. One year later, a follow-up CT scan did not show any abnormalities.

## Case 2

A 73-year-old man underwent percutaneous angioplasty due to unstable angina CCS III-IV. He had a history of mild chest pain for the last 20 years with a crescendo pattern in the early past. No pathological findings were detectable in electrocardiography. His cardiovascular risk factors included history of nicotine abuse, hypertension, as well as hyperlipidemia. Due to the severity of the angina an elective catheterization via the right radial artery was planned. Mecain 1% injection solution was used as a local anesthetic and verapamil was administered as an intra-arterial vasodilatory agent. For anticoagulation 5.000 IE heparin and 150 mg clopidogrel were applied.

The process of advancing the guide wire toward the ascending aorta has been described as difficult due to a kinking of the brachiocephalic trunk. As in the first case, the procedure was performed by an experienced cardiologist with a large number of transradial catheterizations performed. During the procedure the wire was not forced and a polymer-jacketed guidewire was used.

**Figure 2.**
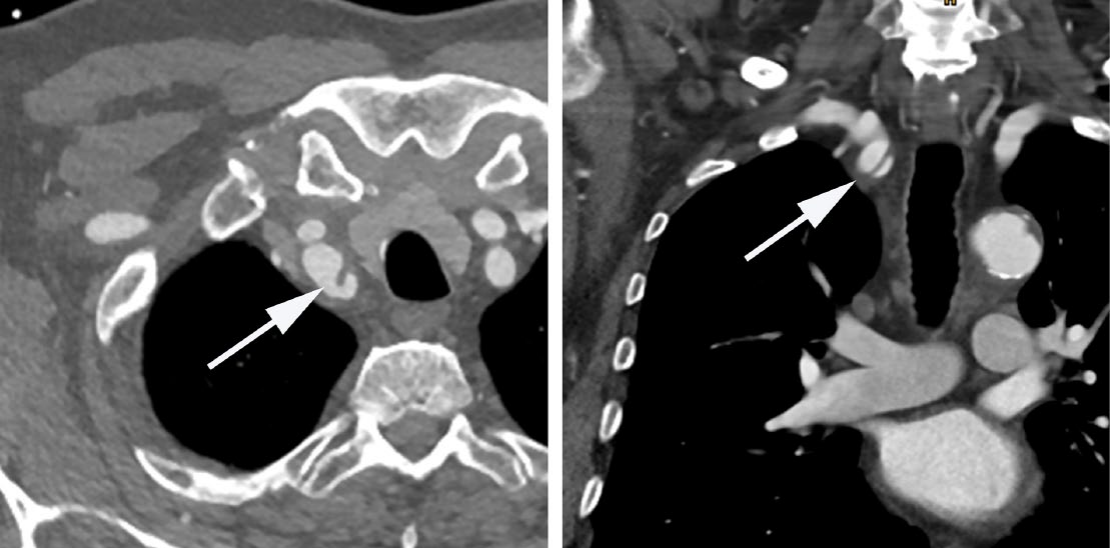
Computed tomography showing dissection of the brachiocephalic trunk (arrows) with sagittal and coronal views.

His coronary angiography revealed the presence of a chronic collateralized stenosis in the proximal third of the right coronary artery, diffuse sclerosis, and calcifications with high degree stenoses of the ramus circumflexus and the proximal left anterior descending coronary artery. After an extensive interdisciplinary discussion of the diagnostic findings it was decided to perform interventional treatment. During the switch to a guide catheter (Braun; Serpia; XB 3,5; 6 French) advancing the guide wire into the ascending aorta again appeared to be difficult and ultimately frustrating. The subsequent angiography demonstrated a strong suspicion of a dissection of the brachiocephalic trunk. A subsequent CT angiography confirmed a dissection of both the brachiocephalic trunk as well as the aortic arch (Figure 2). The patient was referred to the Department of Cardiac Surgery for further conservative treatment and coronary artery bypass grafting. No repair of the ascending aorta was required, as the false lumen of the dissection spontaneously thrombosed and the dissection was treated conservatively.

## Discussion

Coronary angiography is the gold standard as invasive evaluation and management of coronary artery disease. In this work, we describe severe complications of transradial approaches resulting in central vascular dissection and perforation in 2 patients who underwent elective heart catheterization.

An extensive review of literature only revealed a small number of articles describing arterial complications using the transradial access.^5^ To our knowledge, this is the first report delineating a dissection of the brachiocephalic trunk related to heart catheterization via the right radial artery. Moreover, in the past only a few case reports dealing with iatrogenic perforation of a central vessel as a complication of coronary angiography from the radial route were published.

In this regard, Habib et al described a perforation of the right subclavian artery, located between the origin of the right carotid artery and the origin of the right vertebral artery. Previously, despite several attempts, the ascending aorta could not be attained and the guidewire repeatedly entered the descending aorta. The patient remained hemodynamically stable and a covered stent was implanted via the femoral artery.^6^

Additionally, Farooqi et al brought attention to a case describing a perforation of the costocervical trunk complicated by a rapidly expanding cervical hematoma with consecutive airway constriction. After transfemoral coil embolization, the patient recovered and could be discharged home after 10 days.^7^

Finally, Abdool et al reported on an iatrogenic perforation of a tortuous right subclavian artery. After 12 hours of conservative treatment, permanent need for blood transfusions was the clincher for the insertion of a covered stent-graft from the right femoral route into the right subclavian artery. Over a period of 2 weeks the patient made an uneventful recovery.^8^

In the majority of the cases mentioned above, resistance was encountered during the guidewire advancement. Especially the anatomic kinking of the subclavian artery or the brachiocephalic trunk can lead to a difficult positioning of the diagnostic catheter. By means of application of small and hydrophilic guidewires or the appropriate switch to the femoral or contralateral radial access in cases of an unsuccessful passage serious vascular adverse events can be reduced.

Sanmartin and coworkers analyzed all upcoming hemorrhagic complications in their center from a total of 3403 coronary angiographies using radial access. Whereas a perforation of the radial artery occurred in 4 patients, only 1 patient presented a perforation of the brachial artery.^9^

Even though vascular trauma can occur, minimally invasive endovascular treatment may be successfully used for such complications and has been shown to have 96.9% success rate for stent implantation and sufficient stent patency during follow-up.^10^ In general, radial catheterization is as effective as the femoral approach and is associated with additional benefits. Due to the superficial location of the artery, an optimal mechanical control during vascular compression after removal of the catheter sheath is provided. Therefore, bleeding complications after puncture of the radial artery are extremely rare.^11^ Moreover, a direct comparison of both access showed a significantly lower incidence of cardiac or cerebrovascular events and shorter hospital stay in patients managed via the radial artery.^6^ As a result, the transradial approach in general is considered to be safer than the transfemoral approach with an extremely low incidence of complications that were even trivialized in the literature.^12-15^

However, as it can be seen from the present case series, severe complications can still occur even in cases of elective procedures. Therefore, it is of paramount importance to be aware of potential major complications related to the transradial access and to comprehend their prevention and acute management.

Referring to the advantages specified above, cardiac catheter examination via the right radial artery remains the first choice in a number of centers including ours. By the use of small-sized introducer sheaths as well as short examination times, the incidence of vascular occlusions consequently remains at a low level. Moreover, by administering heparin for anticoagulation and intra-arterial calcium antagonists or nitrate for prevention of arterial spasms, we are able to counteract possible complications at an early stage.

Due to a rising life expectancy and increase in the incidence of various risk factors, the management of coronary artery disease plays a vital role in the modern medicine. Therefore, the knowledge of possible complications and their clinical appearance is essential. Although no surgical intervention was necessary in our cases, guidelines enabling saving and quick handling of possible complications are important. Finally, close collaboration of both cardiologists and cardiac surgeons in joint clinical centers can provide the optimal standard of safety and success of treatment as well as an outstanding interdisciplinary management of possible complications.
